# A room-temperature antiferroelectric in hybrid perovskite enables highly efficient energy storage at low electric fields†

**DOI:** 10.1039/d2sc05285g

**Published:** 2022-10-28

**Authors:** Yi Liu, Haojie Xu, Xitao Liu, Shiguo Han, Wuqian Guo, Yu Ma, Qingshun Fan, Xinxin Hu, Zhihua Sun, Junhua Luo

**Affiliations:** State Key Laboratory of Structure Chemistry, Fujian Institute of Research on the Structure of Matter, Chinese Academy of Sciences Fuzhou Fujian 350002 P. R. China sunzhihua@fjirsm.ac.cn jhluo@fjirsm.ac.cn; University of Chinese Academy of Sciences Beijing 100049 P. R. China; Fujian Science & Technology Innovation Laboratory for Optoelectronic Information of China Fuzhou Fujian 350108 P. R. China

## Abstract

Molecular antiferroelectrics (AFEs) have taken a booming position in the miniaturization of energy storage devices due to their low critical electric fields. However, regarding intrinsic competitions between dipolar interaction and steric hindrance, it is a challenge to exploit room-temperature molecular AFEs with high energy storage efficiency. Here, we present a new 2D hybrid perovskite-type AFE, (i-BA)_2_(FA)Pb_2_Br_7_ (1), which shows ultrahigh energy storage efficiencies at room temperature. Most strikingly, the typical double *P*–*E* hysteresis loops afford an ultrahigh storage efficiency up to ∼91% at low critical electric fields (*E*_cr_ = 41 kV cm^−1^); this *E*_cr_ value is much lower than those of state-of-the-art AFE oxides, revealing the potential of 1 for miniaturized energy-storage devices. In terms of the energy storage mechanism, the dynamic ordering and antiparallel reorientation of organic cations trigger its AFE-type phase transition at 303 K, thus giving a large spontaneous electric polarization of ∼3.7 μC cm^−2^, while the increasement of steric hindrance of the organic branched-chain i-BA^+^ spacer cations stabilizes its antipolar sublattices. To the best of our knowledge, this exploration of achieving ultrahigh energy storage efficiency at such a low critical electric field is unprecedented in the AFE family, which paves a pathway for miniaturized energy storage applications.

## Abstract 2

We report a room-temperature molecular AFE, (i-BA)_2_(FA)Pb_2_Br_7_, which shows ultrahigh *η* of ∼91% at quite low electric fields. This finding will be advantageous to the progress of high energy-storage applications.

## Graphical abstract

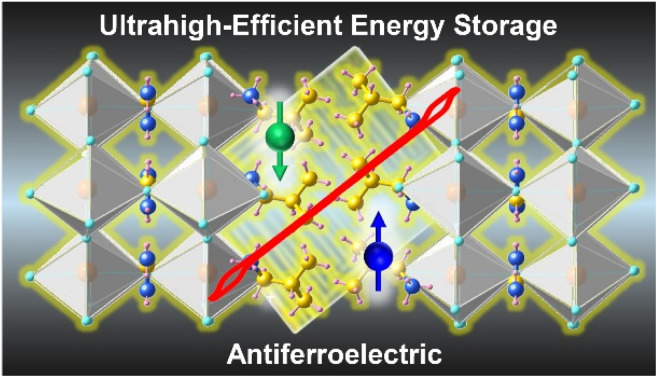

## Introduction

Solid-state energy storage materials have recently attracted increasing attention, due to the highly urgent demand for sustainable energy sources over the past decade.^1–3^ Conceptually, antiferroelectric (AFE) materials, characterized by the antiparallel arrangement of adjacent sublattice polarizations,^4,5^ are emerging as a remarkable candidate for efficient energy-storage applications.^6^ Benefiting from their typical polarization *versus* electric field (*P*–*E*) double hysteresis loops,^7^ the unique field-induced AFE-to-ferroelectric (FE) structural transition leads to prominent energy-storage performances,^8^ including superior energy density, fast charge–discharge rate, and excellent fatigue endurance. These advantages are far beyond linear dielectric and FE materials, as demonstrated by AFE oxides that possess high critical fields (*E*_cr_), such as AgNbO_3_ (*E*_cr_ ∼150 kV cm^−1^), PbHfO_3_ (*E*_cr_ ∼270 kV cm^−1^), and PbZrO_3_ (*E*_cr_ ∼463 kV cm^−1^).^9–11^ By contrast, molecule-based soft AFEs with intrinsic merits of structural flexibility, facile processability and low operating voltage, are booming as alternatives for energy storage.^12–16^ It is emphasized that their low *E*_cr_ guarantees the possibility of high-level integration into miniaturized electronic devices. For most of the known molecular AFEs, however, an obvious drawback is their low energy-storage efficiency (*η*), such as TFMBI (*E*_cr_ ∼22 kV cm^−1^, *η* ∼ 44%) and TCMBI (*E*_cr_ ∼81 kV cm^−1^, *η* ∼ 62%).^17,18^ This is ascribed to the large hysteresis (Δ*E*) between forward and backward switching in the *P*–*E* loops. The low *η* value signifies high energy dissipation that transforms into thermal energy, thus drastically degrading the storage capability. Besides, the optimal *η* and lower *E*_cr_ values usually emerge near the Curie temperature (*T*_c_) for the AFE-to-paraelectric phase transition, for molecular AFEs such as NH_4_H_2_PO_4_ (*T*_c_ = 148 K),^19^ 2-difluoromethylbenzimidazole (*T*_c_ > 413 K)^13^ and cyclohexylmethylammonium bromide (*T*_c_ = 364 K),^20^ thus hindering their potential applications. In this context, it is urgently necessary to assemble new room-temperature AFE candidates that enable high energy-storage efficiency under low electric fields.

Recently, the family of 2D Ruddlesden–Popper hybrid perovskites, showing rich and intriguing physicochemical properties,^21–24^ have provided new entries into diverse applications, such as light-emitting diodes,^25,26^ solar cells,^27,28^ lasers,^29–31^ and photodetectors.^32–35^ The most fascinating virtue of these 2D members is the breakthrough of the structural limitation of the tolerance factor, which permits an infinite possibility for incorporating bulky spacer cations between inorganic sheets.^36^ Dynamic motions or orientations of organic spacers are controllable by applying an external electric field; this will greatly facilitate the generation of molecular dipoles and electric order. Previous studies have concentrated on the mixed-cation alloying of flexible moieties to assemble new FE materials, such as (4,4-difluorohexahydroazepine)_2_PbI_4_,^37^ (isoamylammonium)_2_(EA)_2_Pb_3_Br_10_,^38^ and (4-aminomethyl-1-cyclohexanecarboxylate)_2_(EA)_2_Pb_3_Br_10_.^39^ Contrary to the robust advance of FEs, however, antiferroelectricity in this 2D hybrid perovskite system is still a virgin land with bright prospects. Regarding the antipolar structural and free energy criteria for AFEs, the incorporation of branched-chain organic cations allows a dramatic increasement of steric hindrance in a confined space. Hence, the repulsive interactions caused by the steric hindrance effect would stabilize the antipolar array of dipoles, *i.e*., the formation of the AFE structure. As verified by organic molecular AFEs, the steric hindrance of organic moieties reduces the distance between adjacent dipoles, thereby creating antipolar motifs.^40–42^ Despite continued efforts, it remains a challenge to explore room-temperature molecular AFEs in this 2D family, owing to the lack of knowledge regarding the competition balance between steric hindrance and dipolar interactions in homogeneous sublattices.

Here, we have successfully achieved a room-temperature molecular AFE with ultrahigh energy storage efficiency by alloying the branched-chain cation as an interlayered spacer of 2D multilayered hybrid perovskites, (i-BA)_2_(FA)Pb_2_Br_7_ (1, where i-BA^+^ is iso-butylammonium, and FA^+^ is formamidinium). Strikingly, the characteristic double *P*–*E* hysteresis loops enable ultrahigh energy-storage efficiency of ∼91% under low *E*_cr_ about 41 kV cm^−1^. Such merits reveal its great potential for miniaturized energy-storage devices. The antiparallel reorientations of i-BA^+^ and FA^+^ cations lead to its room-temperature antiferroelectricity with a Curie point (*T*_c_) of 303 K, while the increasement of steric hindrance of i-BA^+^ spacer cations plays an important role in stabilizing its antipolar AFE motif. As far as we know, 1 is the first molecular antiferroelectric to achieve ultrahigh energy storage efficiency at such a low critical electric field, shedding light on exploration of new AFE candidates toward practical applications, *e*.*g*., high energy-storage capacitors.

## Results and discussion

### Variable-temperature structure analyses

**Fig. 1:**
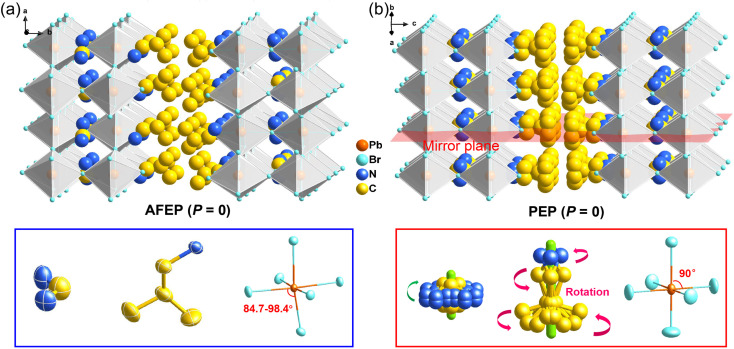
Crystal structure packing diagrams of 1 in the (a) LTP (left) at 200 K and (b) HTP (right) at 310 K. Upon heating, it is obvious that i-BA^+^ and FA^+^ cations become disordered with the dynamic reorientation, while the distorted PbBr_6_ octahedra return to be regular.

Bulk crystals of 1 were prepared from its HBr solution by the slow temperature-cooling method (Fig. S1†), and the purity of the phase was verified by using powder X-ray diffraction (Fig. S2†). Variable-temperature crystal structures of 1 at 200 K (low-temperature phase, LTP) and 310 K (high-temperature phase, HTP) were determined by X-ray diffraction to understand the mechanism of phase transition and the origin of its antiferroelectricity. In the LTP, the structure of 1 is found to be in the orthorhombic space group *Pnma* (Table S1†). As depicted in Fig. 1a, the basic crystal architectures consist of two perovskite slabs of PbBr_6_ octahedra, separated by a thickness of two i-BA^+^ organic cations. A highly contorted perovskite framework verified by the unequal Br–Pb–Br bond angles (84.7–98.4°) allows for the alloying of the FA^+^ cation into perovskite cavities. For the organic components, the FA^+^ and i-BA^+^ cations are ordered and the NH_3_^+^ groups in them are antiparallelly aligned toward the *a*-axis utilizing the N–H⋯Br hydrogen bond interaction with the inorganic framework (Fig. S3†). Such an antiparallel configuration of neighboring dipoles results in an antipolar state (*P*_s_ = 0), which reveals the probable antiferroelectricity of 1.

**Fig. 2:**
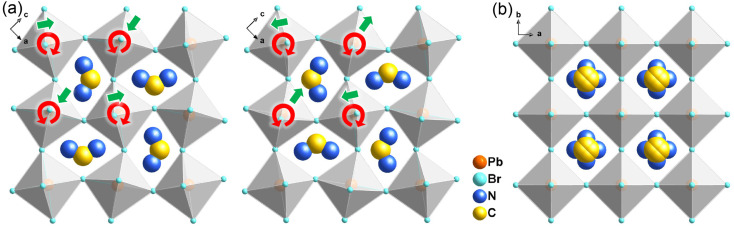
Schematic view of the rotational and tilted motions of PbBr_6_ octahedra between the (a) LTP and (b) HTP of 1. Red and green arrows represent the directions of rotation and tilting, respectively.

At the HTP, 1 crystallizes in the tetragonal centrosymmetric space group *I*4/*mmm*. In terms of polarization, the characteristic of the HTP is that both i-BA^+^ and FA^+^ cations are located on a crystallographic mirror plane with the completely disordered patterns, thus cancelling the net polarization of the crystal (Fig. 1b). The rotational motion of these organic cations is easily activated at around room temperature, indicating the quite small potential energy barrier of 1, which can be conducive to the generation of molecular dipoles and electric order controlled by applying a low external electric field. Meanwhile, the distorted inorganic PbBr_6_ octahedra translate to the highly symmetric configurations. This evolution is driven by both their rotational motion around their respective centers with the rotation axis parallel to the *b* direction and anti-parallel tilting in adjacent inorganic layers (Fig. 2). Therefore, it is the thermally induced order–disorder transition of organic cations and dynamic distortion of inorganic PbBr_6_ octahedra that synergistically result in the room-temperature antiferroelectric-to-paraelectric phase transition behavior of 1 under low electric fields.

#### Phase transition properties

**Fig. 3:**
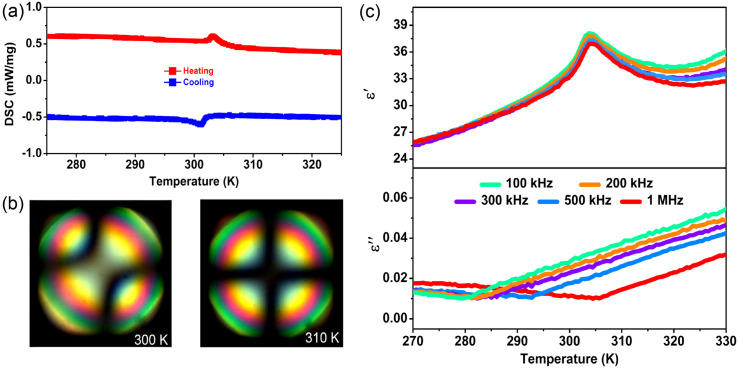
Phase transition properties of 1. (a) DSC curves of 1 measured in the heating and cooling runs. (b) Conoscopic images of the biaxial and uniaxial nematic phases at 300 and 310 K, respectively. (c) The real part (*ε*′) and imaginary part (*ε*′′) of the complex dielectric constant measured along the crystallographic *c*-axis direction at different frequencies upon heating.

To confirm the phase transition behavior of 1, the differential scanning calorimetry (DSC) measurement was first implemented. Fig. 3a shows the DSC curves that clearly exhibit one pair of reversible peak-type thermal anomalies at 303.2/301.3 K (*T*_c_) in the heating/cooling processes, accompanied by a narrow thermal hysteresis of 1.9 K, indicating the feature of the second-order phase transition. As shown in Fig. 3b, conoscopic images have definitely proved the evolution from biaxial nematic phases (at 300 K) to the uniaxial nematic phases (at 310 K), which coincides with the structural phase transition of *mmm*-to-4/*mmm*. Further, we performed the dielectric measurements of 1 in the temperature range 270–330 K. Fig. 3c exhibits the variation of the real part (*ε*′) and imaginary part (*ε*′′) of the complex dielectric constant at selected frequencies. The *ε*′–*T* plots show peak-like anomalies with the maximum around 303 K and the *ε*′ values drop gradually on alternating the frequency from 10^5^ to 10^7^ Hz, suggesting that the dynamic motion of dipoles cannot keep up with the quick switching of the applied electrical field at higher frequencies. The quite small *ε*′′ values of dielectric loss (<0.04) demonstrate the low inherent thermal dissipation of 1, which can improve its working stability.^43,44^ A significant frequency dispersion of the dielectric loss is observed in the temperature region below *T*_c_, that is, the increasing of *T*_c_ at higher frequency. In contrast to the *ε*′–*T* trace, the frequency dispersion becomes more prominent in the dielectric loss plot; this is the characteristic feature of dielectric relaxation behavior. Generally, this relaxation process of dielectric properties dependent on the applied frequency is closely related to the molecular dipole motions or ionic displacements. Considering the order–disorder structural changes of organic i-BA^+^ and FA^+^ cations during the phase transition, it is reasonable to believe that their dipolar motions result in the dielectric relaxation behavior, which would be greatly favorable for the low-loss energy storage of 1.^45–47^

#### Antiferroelectric properties

**Fig. 4:**
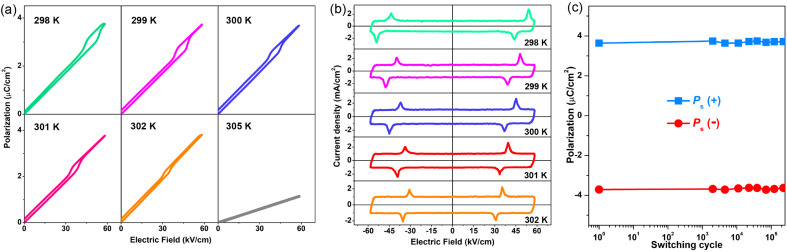
The AFE properties of 1. (a) The *P*–*E* hysteresis loops and (b) corresponding *J*–*E* curves. (c) The fatigue endurance of 1 after ∼2 × 10^5^ switching cycles under low field and room temperature conditions.

**Fig. 5:**
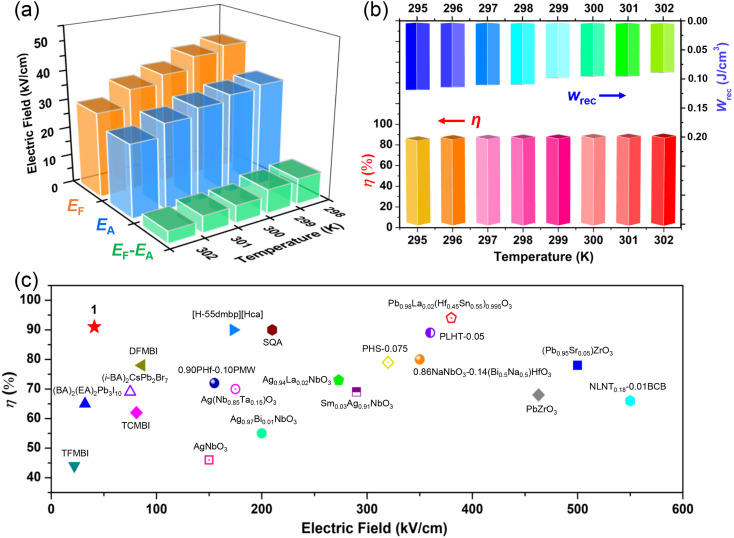
(a) *E*_F_, *E*_A_, and Δ*E* obtained at different temperatures. (b) Temperature dependence of energy density *W*_rec_ and storage efficiency *η*. (c) Energy storage efficiency (*η*) for various reported antiferroelectrics at room temperature.

Generally, the key feature of AFE materials is that the antiparallel arrangement of adjacent dipoles in the sublattices causes zero *P*_s_ (antipolar state) under the zero electric field. These molecular dipoles can be reversibly reversed and switched to be parallel under a sufficiently strong external field. This evolution process represents the electric field-triggered AFE-to-FE phase transition (phase switching) and is typically identified by the double *P*–*E* hysteresis loops. As shown in Fig. 4a, the polarization response of 1 to the electric field is linear at 305 K in the HTP, which is similar to that of ordinary dielectric materials. With the temperature just decreasing below *T*_c_, the typical double *P*–*E* hysteresis loops become conspicuous, showing the assured proof of antiferroelectricity. The *P*_max_ value is calculated to be ∼3.7 μC cm^−2^ under a low electric field of 41 kV cm^−1^ at room temperature, and the *P*–*E* loops change to be slightly thinner with the increasing of temperature. Such low electric fields guarantee the reliability of operation, thus, providing the possibility of high-level integration of miniaturized electronic devices. In addition, this *P*_s_ value is on par with those of some other molecular AFE compounds, such as 2-difluoromethylbenzimidazole (∼6 μC cm^−2^),^13^ (BA)_2_(EA)_2_Pb_3_I_10_ (∼5.6 μC cm^−2^),^15^ (3-pyrrolinium)CdBr_3_ (∼5.6 μC cm^−2^),^16^ 2-trifluoromethylbenzimidazole (∼5.9 μC cm^−2^),^18^*etc*. From a structural viewpoint, the generation of polarization closely involves the dynamic ordering and antiparallel reorientations of FA^+^ and i-BA^+^ cations. The low *P*_s_ result of 1 may be caused by the inherent symmetry and orientation of the FA^+^ cations, being similar to those of the 2D hybrid perovskite ferroelectric (BA)_2_(FA)Pb_2_Br_7_ (∼3.8 μC cm^−2^).^57^Fig. 4b illustrates the representative current–field (*J*–*E*) traces of 1. Clearly, there are sharp peaks of current observed during the electric-induced phase transitions between the AFE and FE states. The current density exhibits a temperature-dependent behavior, which coincides well with that of the *P*_s_–*T* variation. The double *P*–*E* hysteresis loops and *J*–*E* traces present the frequency stability over the frequency range of 40–100 Hz (Fig. S4†). Furthermore, we study the fatigue endurance of antiferroelectric materials. Fig. 4c shows that *P*_s_ of 1 remains unchanged after 2 × 10^5^ switching operation cycles at low field and room temperature conditions. Such an excellent fatigue characteristic makes 1 a promising candidate toward the possible application in miniaturized energy storage devices. Besides, the forward switching field (*E*_F_, AFE-to-FE transition) and backward switching field (*E*_A,_ FE-to-AFE transition) were also acquired from these *J*–*E* traces of 1. Fig. 5a presents the evolution of *E*_F_, *E*_A_, and the field hysteresis Δ*E* (Δ*E* = *E*_F_ − *E*_A_), all of which decrease monotonically with the increasing temperature, and the Δ*E* is estimated to be 4.2 kV cm^−1^ at 302 K. As far as we know, this Δ*E* value for 1 is much smaller than that for other hybrid AFEs and inorganic AFE ceramics, such as (i-BA)_2_CsPb_2_Br_7_ (∼47 kV cm^−1^),^14^ Ag_0.94_La_0.02_NbO_3_ (∼75 kV cm^−1^),^48^ (Pb_0.95_Sr_0.05_)ZrO_3_ (∼190 kV cm^−1^),^49^*etc.* This unique advantage would effectively reduce the energy loss and probably cause remarkable energy storage efficiency (*η*).

#### Energy storage properties

All the above studies confirm that 1 is a room-temperature molecule-based AFE material in the family of 2D hybrid perovskites. The energy storage efficiency (*η*) and recoverable energy density (*W*_rec_) are two important figures-of-merit to appraise the energy storage performances for practical applications. Here, we have studied temperature dependence of *η* and *W*_rec_ of 1, as deduced from its double *P*–*E* loops with the following equations: *η* = *W*_rec_/(*W*_rec_ + *W*_loss_) and 
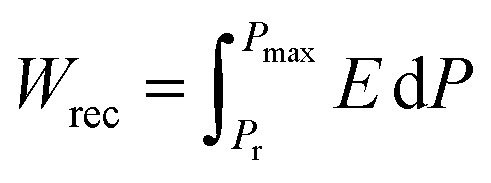
, where *E* is the applied electric field, *P*_r_ and *P*_max_ are the remnant polarization and maximum polarization, respectively, and *W*_loss_ represents energy loss (green areas in Fig. S5†). At room temperature, the *η* and *W*_rec_ values are estimated to be ∼89% and 0.13 J cm^−3^ from the double *P*–*E* hysteresis loop. On increasing the temperature, a satisfactory thermal stability has been confirmed, with the variations of both *W*_rec_ and *η* being less than 5%. It is fascinating that the temperature dependence of *η* shows a gradual increase up to ∼91% at 302 K, due to the decrease of *W*_loss_ caused by the shrinking of *P*–*E* loops (Fig. 5b). From the perspective of practical application, the frequency dependence of energy storage performances is also of great significance. With the frequency decreasing from 100 to 40 Hz (at 302 K, 41 kV cm^−1^), the *P*_max_ value slightly decreases, while the Δ*E* increases (Fig. S4†). This indicates the energy dissipation of 1 remains at a low level, thus accounting for the stability of *W*_rec_ and *η* (Fig. S6†). As far as we know, this energy storage efficiency is even higher than that of the majority of inorganic AFE ceramics, such as Hf_0.3_Zr_0.7_O_2_ (*η* ∼ 50%),^50^ PHS-0.075 (*η* ∼ 79%),^51^ and PLHT-0.05 (*η* ∼ 89%) (Fig. 5c and Table S5†).^52^ The high energy storage efficiency signifies the low energy dissipation, which will less easily transform into thermal energy, thus drastically promoting its energy storage capacity. Such an ultrahigh energy efficiency at room temperature, together with the low critical fields, demonstrates the great potential of 1 for the application of energy storage with high practicability. In terms of improving the *W*_rec_, the chemical design of organic cations, such as H/D substitution, plays an important role in increasing the *T*_c_ and the *P*_s_ of molecular ferroelectrics, which provides a hint for the realization of high-performance molecular antiferroelectric candidates.^53^ Besides, ion doping, including halogen anions or skeleton cations, may be an effective approach to increase the *W*_rec_ due to the introduction of relaxor characteristics.^45^

#### Origin of energy storage performance

**Fig. 6:**
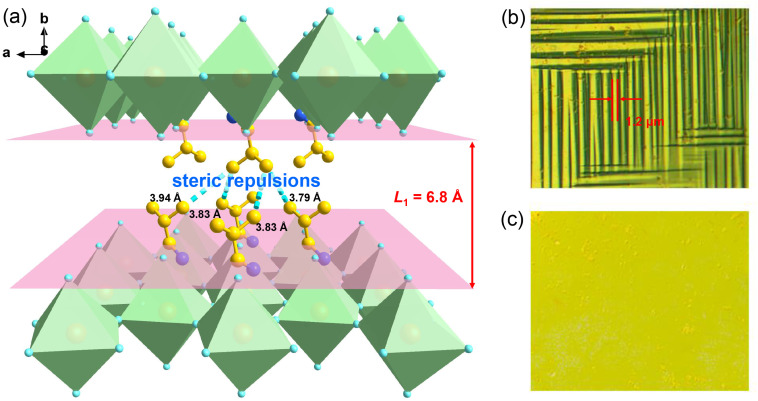
(a) The interlayered space and atomic distance between the adjacent layers of organic i-BA^+^ cations in 1. Time evolution of domain patterns observed at different temperatures: (b) 300 K and (c) 310 K.

To better understand the spacer cation arrangement that controls the tendency toward ferroelectric or AFE ordering, we have taken the typical examples of (*n*-BA)_2_(FA)Pb_2_Br_7_ and (i-BA)_2_(FA)Pb_2_Br_7_ (1) for a detailed analysis. It is understood that the length and bulkiness of spacer cations play an important role in the formation of 2D perovskite frameworks, described by the distance between two adjacent inorganic sheets. As depicted in Fig. 6a, this plane distance is estimated to be ∼6.8 Å for 1, while the corresponding value for (*n*-BA)_2_(FA)Pb_2_Br_7_ containing the linear *n*-BA^+^ cation is ∼7.5 Å (Fig. S7a†). Hence, the reduction of space distance between two adjacent inorganic perovskite layers for 1 suggests the stronger confinement effects imposed by the rigid scaffold, which may probably influence the competition between their dipolar interactions and steric hindrance. That is, smaller distance between the adjacent parallel dipoles leads to more significant steric repulsions. As shown in Fig. S7b,† since the bulky tails of i-BA^+^ cations raise the steric hindrance, the average distance between the neighboring organic layers is about 3.85 Å, which becomes shorter by approximately 13% than that of *n*-BA^+^ (∼4.41 Å), revealing a stronger geometric constraint in 1. Consequently, the antipolar arrangement of structural moieties is also influenced by steric repulsions, which facilitate their stable AFE crystalline sublattices with the antiparallel array (Fig. S8†). Such findings demonstrate that our strategy of incorporating the branched-chain alkylamine i-BA into the interlayered space of 2D hybrid perovskites is viable for the generation of antiferroelectric order.

Further, we employed polarized light microscopy techniques to investigate the domain structure of 1, in order to better understand the microscopic origin of its excellent energy storage performance. Under perpendicular polarized light, the domains of different orientations possess different birefringence properties, thus exhibiting dark and bright patterns. As depicted in Fig. 6b and c, the crystalline sheets of 1 exhibit two types of periodic stripe domain walls with an average width ≈1.2 μm in the LTP, which are almost perpendicular to each other. In general, such domains of micrometer scale decrease the polarization hysteresis between the AFE–FE and FE–AFE phase transitions owing to the fast response of microscale domains to the applied electric field, induced by the weak interatomic interactions between the microdomain clusters.^46,54–56^ Consequently, a slim hysteresis loop with high energy storage efficiency can be acquired by regulating the domain size to the micrometer scale in AFEs. As the temperature increases to the HTP, the domain structures slowly disappear, corresponding to its paraelectric state. Subsequently, as the temperature cools down to the LTP, the crystalline sheet transforms back into the ferroelastic phase with the reconstructed domain structures. Such a temperature dependence of the domain patterns also coincides well with the AFE-to-paraelectric phase transition of 1. Furthermore, it is known that the formation of solid solutions with relaxor compounds is an efficiency-enhancement strategy for antiferroelectric ceramics with relaxor characteristics, as demonstrated in some inorganic antiferroelectric counterparts. The lattice distortion and reduction in the grain size are also observed for relaxor compound additions, which will lead to changes in the domain structure and size. It is proposed that nanosized domains characteristic of relaxor-antiferroelectrics can be achieved in 2D hybrid perovskite materials through constructing solid solutions using halogen doping methods.^46,53^

## Conclusions

In summary, we have successfully designed a new 2D hybrid perovskite-type molecular AFE, (i-BA)_2_(FA)Pb_2_Br_7_, which demonstrates an ultrahigh energy storage efficiency under low electric fields at room temperature. Structurally, the dynamic ordering and antiparallel reorientation of organic dipoles induce its AFE-type phase transition at 303 K along with a large *P*_s_ of ∼3.7 μC cm^−2^. It is noteworthy that the antipolar sublattices of 1 have been stabilized through raising steric hindrance of organic i-BA^+^ spacer cations. Most strikingly, the characteristic double hysteresis loops of 1 allow a high energy storage efficiency ∼91% at a quite low *E*_cr_ ∼41 kV cm^−1^. This *E*_cr_ value is much lower than those of state-of-the-art AFE oxides, revealing its great potential for the miniaturization of energy-storage devices. This work sheds light on exploration of new AFE candidates toward practical applications.

## Author contributions

Y. L. prepared the samples, measured the antiferroelectric properties, and wrote the manuscript. H. J. X., X. T. L., and S. G. H. determined the structures. W. Q. G., Y. M., Q. S. F., and X. X. H. provided suggestions for research. Z. H. S. and J. H. L. designed and directed the studies. All authors contributed to writing and reviewing the manuscript.

## Conflicts of interest

The authors declare no conflict of interest.

## Supplementary Material

SC-013-D2SC05285G-s001

SC-013-D2SC05285G-s002
