# Supplementary material for: A room-temperature antiferroelectric in hybrid perovskite enables highly efficient energy storage at low electric fields
Source: Chem Sci. 2022 Oct 28;13(45):13499–506. doi: 10.1039/d2sc05285g (PMC9682916; doi:10.1039/d2sc05285g)
Supplement: SC-013-D2SC05285G-s001 [file SC-013-D2SC05285G-s001.pdf]

Electronic Supplementary Material (ESI) for Chemical Science.
This journal is © The Royal Society of Chemistry 2022

Supporting Information

Room-Temperature Antiferroelectric in Hybrid Perovskite Enables Highly-Efficient Energy Storage at Low Electric Fields

Yi Liu,[1] Haojie Xu,[12] Xitao Liu,[1] Shiguo Han,[1] Wuqian Guo,[12] Yu Ma,[12] Qingshun Fan,[12] Xinxin Hu,[12] Zhihua Sun,*[123] and Junhua Luo*[12]

[1]State Key Laboratory of Structure Chemistry, Fujian Institute of Research on the Structure of Matter, Chinese Academy of Sciences, Fuzhou, Fujian, 350002, P. R. China
[2]University of Chinese Academy of Sciences Beijing, 100049, P. R. China
[3]Fujian Science & Technology Innovation Laboratory for Optoelectronic Information of China, Fuzhou, Fujian 350108, P. R. China

Experimental Section

Synthesis and single-crystal growth. The target compound **1** was prepared by mixing stoichiometric ratio of isobutylammonium (10 mmol, 1.52 g), formamidinium acetate (5 mmol, 0.38 g) and $Pb(CH_3COO)_2{\cdot}3H_2O$ (10 mmol, 3.79 g) in the 40% w/w solution of hydrobromic acid. After the continuous stirring for 30 min at 373 K, a clear yellow solution was obtained. Plate-like yellow crystals of **1** were obtained by the temperature cooling method after about three weeks, as shown in Figure S1.

Single-Crystal X-ray Crystallography and Powder X-ray Diffraction. We used the Bruker D8 Quesr/Venture diffractometer with the Mo $K\alpha$ radiation (λ = 0.77 Å) to record X-ray diffraction data. The structures were solved by direct methods and confirmed by the full-matrix least-squares refinements on F^2 using the *SHELXTL* software packing. All non-H atoms were refined anisotropically, and all H atoms were generated by geometrical method and refined by using a "riding" model with $Uiso$ = 1.2 Ueq (C). The above-mentioned structure solution and refinement were conducted in the *Olex*2 software. Crystal data for **1** at 200 and 310 K are listed in Table S1. Powder X-ray diffractometry (PXRD) data was recorded on the MiniFlex 600 X-ray diffractometer equipped with a Cu $K\alpha$ radiation.

Measurements. The dielectric analyses were performed on TongHui TH2828 analyzer in the temperature range of 270-330 K. Single-crystal plates of **1** with the surface deposited by silver conduction paste were used for dielectric measurements. DSC measurement of **1** was recorded by using a NETZSCH DSC 200F3 instrument in the

temperature range of 270-330 K. The powder samples that placed in aluminum crucibles were heated and cooled with a rate of 5 K·min^{-1} under a nitrogen atmosphere. The *P-E* hysteresis loops were carried out on a ferroelectric analyzer (Radiant Precision Premier II). For the *P-E* loop measurement, the crystal sample with size of 0.5 × 0.4 × 0.4 mm^3 was used. The sample thickness was 0.4 mm and both sides were printed with silver electrodes with an area of approximately 0.16 mm^2. In order to avoid electric discharge at high electric field, single crystal of **1** was immersed in silicone oil to measure the *P-E* hysteresis loops. Domain structures of the grown crystal were observed using a Nikon Eclipse LV100POL polarizing microscope along [010] crystal wafer.

[CCDC 2157694 and 2157696 contain the supplementary crystallographic data for this paper. These data can be obtained free of charge from The Cambridge Crystallographic Data Centre via www.ccdc.cam.ac.uk/data_request/cif.]

Figure

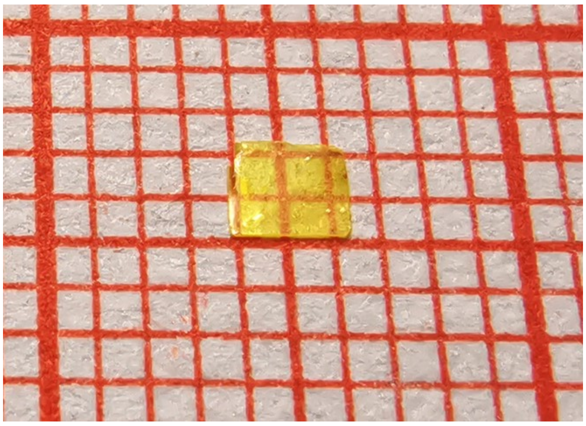

Figure S1. Plate-like single crystal of **1** obtained by the temperature-cooling method.

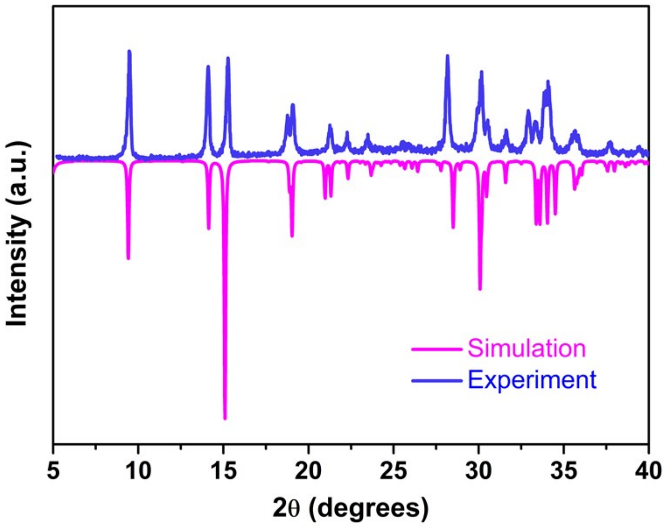

Figure S2. Experimental and simulated PXRD patterns for **1** at room temperature.

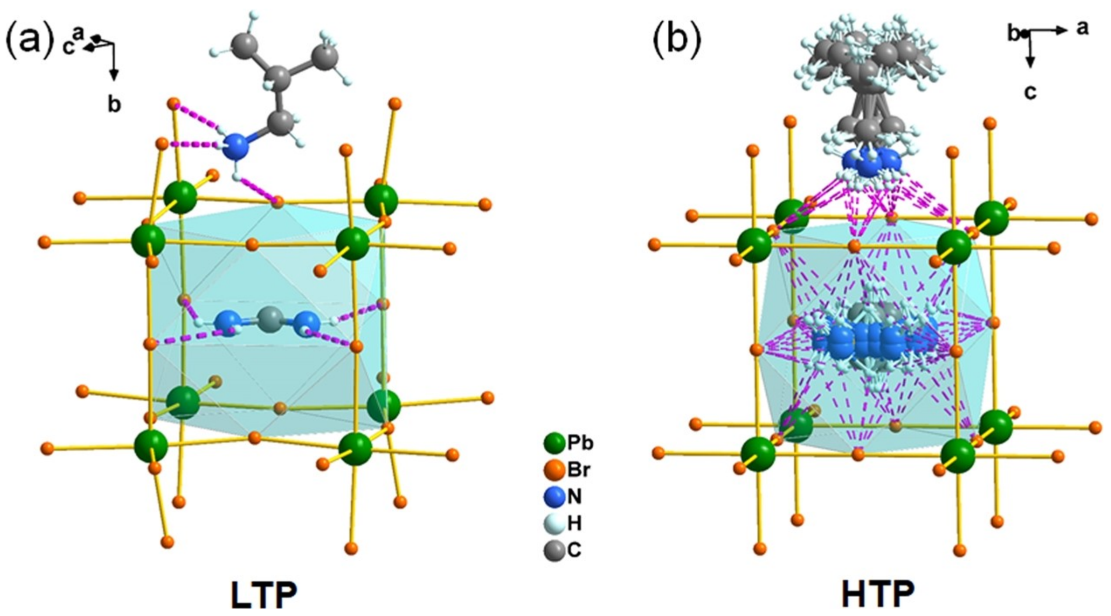

Figure S3. The N-H···Br hydrogen-bonding interactions between organic cations and inorganic perovskite frameworks of **1** at (a) low-temperature phase (LTP, 200 K) and (b) high-temperature phase (HTP, 310 K). Purple dotted lines represent the N-H···Br hydrogen bonds.

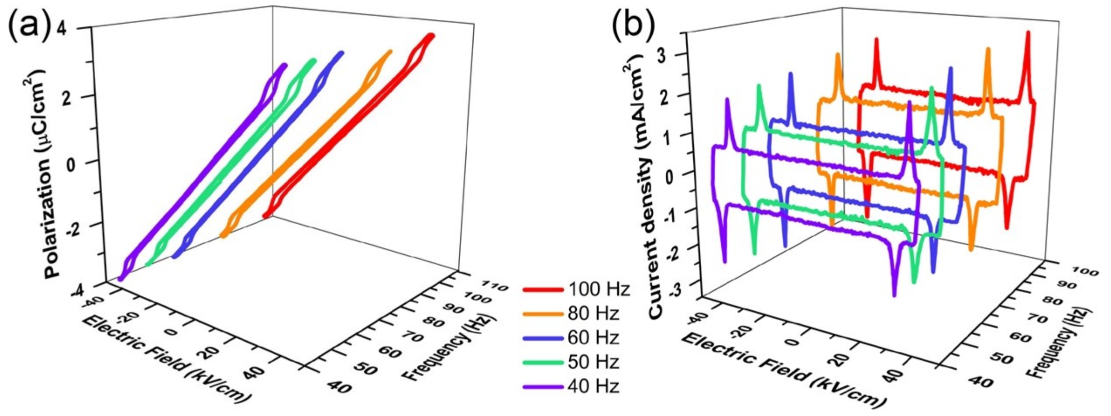

Figure S4. Frequency-dependent (a) double *P-E* hysteresis loops and (b) *J-E* curves.

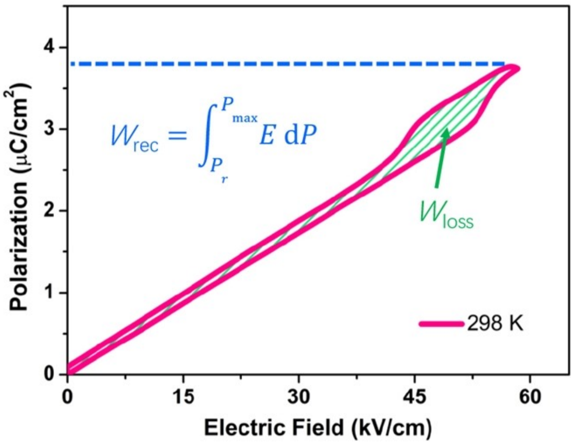

Figure S5. Schematic diagram for energy storage properties of **1** obtained from the typical *P*-*E* hysteresis loop.

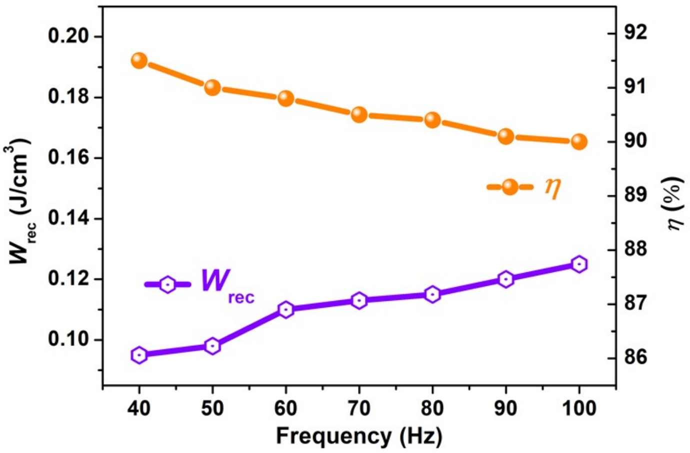

Figure S6. Energy density W_{rec} and energy storage efficiency η of **1** as a function of frequency.

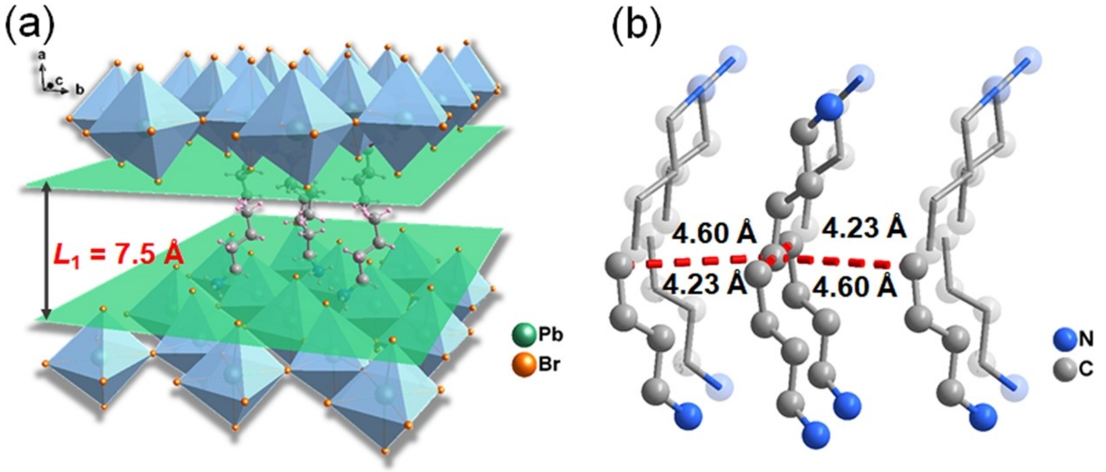

Figure S7. (a) The interlayered space and (b) atomic distance between the adjacent layers of organic *n*-BA^+ cations in (*n*-BA)$_2$(FA)Pb_2Br_7.

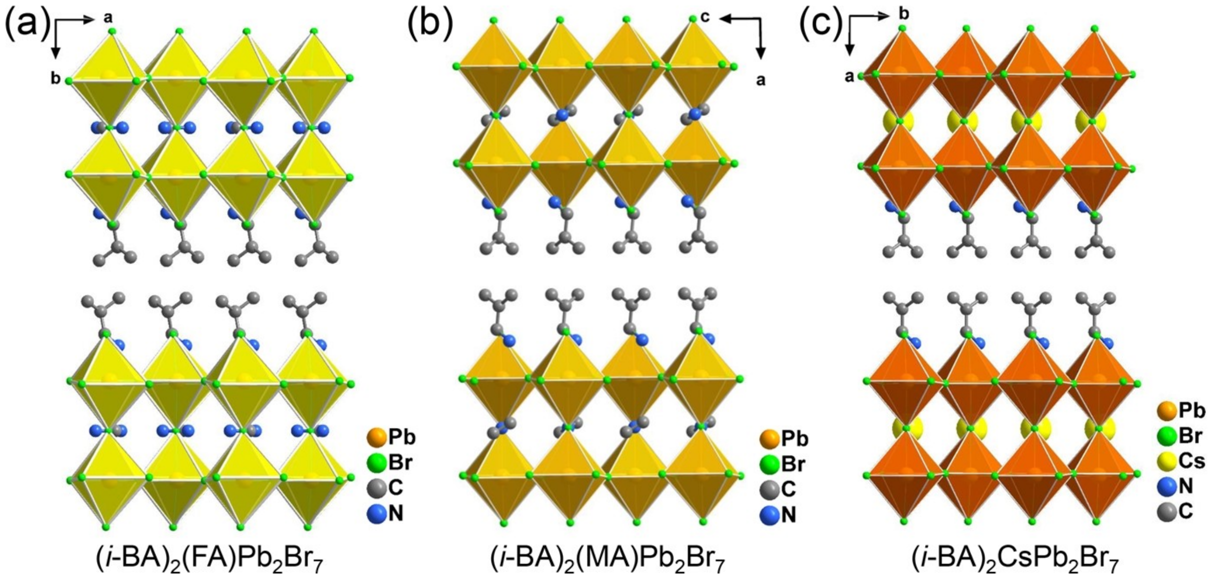

Figure S8. A series of 2D hybrid perovskites, (*i*-BA)$_2$(A)Pb_2Br_7, created by alloying the cationic spacer (*i*-BA$^+$) and "perovskitizer" (FA^+, MA^+ and Cs^+). Obviously, the adjacent *i*-BA$^+$ cations exhibit the antiparallel reorientation.

Table

Table S1. Crystal data for **1** collected at low-temperature phase (LTP, 200 K) and high-temperature phase (HTP, 310 K).

	LTP	HTP
Empirical formula	$C_9H_{29}Br_7N_4Pb_2$	$C_9H_{29}Br_7N_4Pb_2$
Formula weight	1167.11	1167.11
Temperature/K	200	310
Crystal system	Orthorhombic	Tetragonal
Space group	*Pnma*	*I*4/*mmm*
a (Å)	8.3299(4)	5.9841(2)
b (Å)	37.549(3)	5.9841(2)
c (Å)	8.4639(5)	38.4072(17)
V (Å^3)	2647.3(3)	1659.7(3)
D_{calca}/Mg·m^{-3}	2.928	2.818
Z	4	2
μ (mm^{-1})	23.269	22.394
F(000)	2080.0	1040.0
2Θ range /°	4.934 to 54.996	7.03 to 55.17
Index ranges	$-10 \le h \le 10$, $-48 \le k \le 43$, $-10 \le l \le 10$	$-7 \le h \le 7$, $-7 \le k \le 7$, $-49 \le l \le 47$
Reflections collected	21033	5626
Independent reflections	3070 [R_{int} = 0.0758, R_{sigma} = 0.0517]	538 [R_{int} = 0.0649, R_{sigma} = 0.0318]
Data/restraints/parameters	3070/38/109	538/98/88
Goodness-of-fit on F^2	1.083	1.082
Final *R* indexes [$I \ge 2\sigma$ (I)]	R_1 = 0.0554, wR_2 = 0.1474	R_1 = 0.0386, wR_2 = 0.1033
Final *R* indexes [all data]	R_1 = 0.0774, wR_2 = 0.1605	R_1 = 0.0466, wR_2 = 0.1119

Table S2. Bond lengths of crystal **1** at 200 K.

Atom	Atom	Length/Å	Atom	Atom	Length/Å
Pb1	Br1	3.070(5)	N3	C3	1.508(17)
Pb1	Br2^1	2.968(12)	C3	C4	1.44(2)
Pb1	Br2	3.013(12)	C4	N5	1.44(3)
Pb1	Br3^2	3.027(12)	C4	C6	1.51(2)
Pb1	Br3	3.052(12)	N1	C1	1.308(3)
Pb1	Br4	2.916(16)	N2	C1	1.285(2)

Symmetry transformations used to generate equivalent atoms: ^{1}1/2+X, Y, -Z-3/2; ^{2}1/2+X, Y, -Z-1/2

Table S3. Bond angles of crystal **1** at 200 K.

**Bond**	**Angle/°**	**Bond**	**Angle/°**
Br2^{1}-Pb1-Br1	88.85(4)	Br4-Pb1-Br2	98.43(4)
Br2-Pb1-Br1	90.48(4)	Br4-Pb1-Br3^{2}	84.74(4)
Br2^{1}-Pb1-Br2	90.21(13)	Br4-Pb1-Br3	92.64(4)
Br2^{1}-Pb1-Br3	176.31(4)	Pb1^{3}-Br1-Pb1	177.11(6)
Br2-Pb1-Br3	91.75(3)	Pb1^{4}-Br2-Pb1	159.73(5)
Br2^{1}-Pb1-Br3^{2}	90.02(3)	Pb1^{5}-Br3-Pb1	160.25(5)
Br2-Pb1-Br3^{2}	176.82(4)	C4-C3-N3	115.5(12)
Br3-Pb1-Br1	88.00(4)	C3-C4-C6	110.5(15)
Br3^{2}-Pb1-Br1	86.35(4)	C5-C4-C3	118.0(18)
Br3^{2}-Pb1-Br3	87.86(13)	C5-C4-N6	110.5(16)
Br4-Pb1-Br1	171.04(4)	N2-C1-N1	124(3)
Br4-Pb1-Br2^{1}	90.16(4)		

Symmetry transformations used to generate equivalent atoms: ^{1}1/2+X, Y, -Z-3/2; ^{2}1/2+X, Y, -Z-1/2; ^{3}X, -Y+1/2, Z; ^{4}X-1/2, Y, -Z-3/2; ^{5}X-1/2, Y, -Z-1/2

Table S4. Hydrogen bonds of crystal **1** at 200 K.

**D-H···A**	**d(D-H)**	**d(H···A)**	**< DHA**	**d(D..A)**
N1-H1A···Br1^{6}	0.880	3.117	108.60	3.480
N1-H1B···Br1^{1}	0.884	3.128	110.40	3.535
N2-H2C···Br1^{4}	0.880	2.695	142.58	3.436
N2-H2D···Br1^{5}	0.878	2.690	148.83	3.471
N3-H3A···Br4^{1}	0.914	2.499	170.96	3.404
N3-H3B···Br4^{2}	0.908	2.580	164.15	3.463
N3-H3C···Br2^{3}	0.908	2.878	121.28	3.485

Symmetry transformations used to generate equivalent atoms: ^{1}X-1/2, Y, -Z-1/2; ^{2}X-1, Y, Z; ^{3}X-1/2, Y, -Z-3/2; ^{4}X+1/2, Y, -Z-1/2; ^{5}X, Y, Z+1; ^{6}X+1/2, -Y+3/2, -Z+3/2

Table S5. Energy storage efficiency (*η*) for various reported antiferroelectrics at room temperature.

Antiferroelectrics	E_{cr} (kV/cm)	η (%)	Ref.
TFMBI	22	44	[1]
$(BA)_2(EA)_2Pb_3I_{10}$	32	65	[2]
$(i\text{-}BA)_2CsPb_2Br_7$	75	69	[3]
TCMBI	81	62	[1]
DFMBI	86	78	[1]
$AgNbO_3$	150	46	[4]
0.90PHf-0.10PMW	155	72	[5]
[H-55dmbp][Hca]	173	90	[6]
$Ag(Nb_{0.85}Ta_{0.15})O_3$	175	69	[7]
$Ag_{0.97}Bi_{0.01}NbO_3$	200	55	[8]
SQA	210	90	[9]
$Ag_{0.94}La_{0.02}NbO_3$	273	73	[10]
$Sm_{0.03}Ag_{0.91}NbO_3$	290	69	[11]
PHS-0.075	320	79	[12]
$0.86NaNbO_3\text{-}0.14(Bi_{0.5}Na_{0.5})HfO_3$	350	80	[13]
PLHT-0.05	360	89	[14]
$Pb_{0.98}La_{0.02}(Hf_{0.45}Sn_{0.55})_{0.995}O_3$	380	94	[15]
$PbZrO_3$	463	68	[16]
$(Pb_{0.95}Sr_{0.05})ZrO_3$	500	78	[16]
$NLNT_{0.18}\text{-}0.01BCB$	550	66	[17]
**Compound 1**	41	91	This work

References

1. S. Horiuchi, F. Kagawa, K. Hatahara, K. Kobayashi, R. Kumai, Y. Murakami and Y. Tokura, *Nat. Commun.*, 2012, **3**, 1308.

2. S. Han, X. Liu, Y. Liu, Z. Xu, Y. Li, M. Hong, J. Luo and Z. Sun, *J. Am. Chem. Soc.*, 2019, **141**, 12470-12474.

3. Z. Wu, X. Liu, C. Ji, L. Li, S. Wang, Y. Peng, K. Tao, Z. Sun, M. Hong and J. Luo, *J. Am. Chem. Soc.*, 2019, **141**, 3812-3816.

4. Y. Tian, L. Jin, H. Zhang, Z. Xu, X. Wei, E. D. Politova, S. Y. Stefanovich, N. V. Tarakina, I. Abrahams and H. Yan, *J. Mater. Chem. A*, 2016, **4**, 17279-17287.

5. P. Gao, Z. Liu, N. Zhang, H. Wu, A. A. Bokov, W. Ren and Z.-G. Ye, *Chem. Mater.*, 2019, **31**, 979-990.

6. S. Horiuchi, R. Kuma and S. Ishibashi, *Chem. Sci.*, 2018, **9**, 425-432.

7. L. Zhao, Q. Liu, J. Gao, S. Zhang and J.-F. Li, *Adv. Mater.*, 2017, **29**, 1701824.

8. Y. Tian, L. Jin, H. Zhang, Z. Xu, X. Wei, G. Viola, I. Abrahams and H. Yan, *J. Mater. Chem. A*, 2017, **5**, 17525-17531.

9. S. Horiuchi and S. Ishibashi, *Chem. Sci.*, 2021, **12**, 14198-14206.

10. J. Gao, Y. Zhang, L. Zhao, K.-Y. Lee, Q. Liu, A. Studer, M. Hinterstein, S. Zhang and J.-F. Li, *J. Mater. Chem. A*, 2019, **7**, 2225-2232.

11. N. N. Luo, K. Han, F. P. Zhuo, C. Xu, G. Z. Zhang, L. J. Liu, X. Y. Chen, C. Z. Hu, H. F. Zhou and Y. Z. Wei, *J. Mater. Chem. A*, 2019, **7**, 14118-14128.

12. P.-Z. Ge, X.-G. Tang, K. Meng, X.-X. Huang, S.-F. Li, Q.-X. Liu and Y.-P. Jiang, *Chem. Eng. J.*, 2022, **429**, 132540.

13. Z. T. Yang, H. L. Du, L. Jin, Q. Y. Hu, H. Wang, Y. F. Li, J. F. Wang, F. Gao and S. B. Qu, *J. Mater. Chem. A*, 2019, **7**, 27256-27266.

14. P.-Z. Ge, X.-G. Tang, K. Meng, X.-X. Huang, Q.-X. Liu, Y.-P. Jiang, W.-P. Gong and T. Wang, *Mater. Today Phys.*, 2022, **24**, 100681.

15. W. Chao, T. Yang and Y. Li, *J. Mater. Chem. C*, 2020, **8**, 17016-17024.

16. X. Hao, J. Zhai and X. Yao, *J. Am. Ceram. Soc.*, 2009, **92**, 1133-1135.

17. J. Chen, H. Qi and R. Zuo, *ACS Appl. Mater. Interfaces*, 2020, **12**, 32871-32879.
